# Multimodality Fusion Aspects of Medical Diagnosis: A Comprehensive Review

**DOI:** 10.3390/bioengineering11121233

**Published:** 2024-12-05

**Authors:** Sachin Kumar, Sita Rani, Shivani Sharma, Hong Min

**Affiliations:** 1 Akian College of Science and Engineering, American University of Armenia, Yerevan 0019, Armenia; 2 Department of Computer Science and Engineering, Guru Nanak Dev Engineering College, Ludhiana 141006, India; sitasaini80@gmail.com; 3 Department of Computer Science and Engineering, Thapar Institute of Engineering and Technology, Patiala 147004, India; shivani.sharma@thapar.edu; 4 School of Computing, Gachon University, Seongnam 13120, Republic of Korea

**Keywords:** multimodal diagnosis, multimodal fusion, multimodal challenges

## Abstract

Utilizing information from multiple sources is a preferred and more precise method for medical experts to confirm a diagnosis. Each source provides critical information about the disease that might otherwise be absent in other modalities. Combining information from various medical sources boosts confidence in the diagnosis process, enabling the creation of an effective treatment plan for the patient. The scarcity of medical experts to diagnose diseases motivates the development of automatic diagnoses relying on multimodal data. With the progress in artificial intelligence technology, automated diagnosis using multimodal fusion techniques is now possible. Nevertheless, the concept of multimodal medical diagnosis is still new and requires an understanding of the diverse aspects of multimodal data and its related challenges. This review article examines the various aspects of multimodal medical diagnosis to equip readers, academicians, and researchers with necessary knowledge to advance multimodal medical research. The chosen articles in the study underwent thorough screening from reputable journals and publishers to offer high-quality content to readers, who can then apply the knowledge to produce quality research. Besides, the need for multimodal information and the associated challenges are discussed with solutions. Additionally, ethical issues of using artificial intelligence in medical diagnosis is also discussed.

## 1. Introduction

Medical diagnosis entails determining a disease utilizing symptoms, clinical records, and radiological images. Modality, referring to the method used in medical diagnosis, can be categorized as either single or multimodal. By employing a single-modality approach, medical experts use one technique to diagnose the disease, whether through the patient’s symptoms, clinical records, or medical imaging. Multimodal medical diagnosis refers to the use of multiple modalities in medical diagnosis. However, the growing number of patients and the limited availability of expert physicians and specialists in hospitals especially in rural and remote areas have led to the adoption of artificial intelligence (AI).

In developing countries, the disparity between urban and rural health services is a serious problem, with the lack of qualified health care providers being the main cause of the unavailability and low quality of health care in rural areas. Medical AI technology could not only improve the efficiency of doctors and the quality of medical services, but other health workers could also be trained to use this technology to compensate for the shortage of doctors, thereby improving the availability of medical care and the quality of medical services in rural areas.

AI has been extensively used in medical diagnosis and has demonstrated its potential in disease classification and prognosis. However, the majority of current applications of artificial intelligence (AI) in medical diagnosis are constrained to single-modality data. Researchers have extensively studied early detection for various medical conditions in patients utilizing MRI, radiology, CT scans, clinical records, and other techniques. While these models are highly accurate, there are cases where they are not reliable, such as when imaging methods fail to identify a disease while clinical records indicate the presence of an infection or a disease growth, and vice versa. Therefore, multimodal medical diagnosis offers a reliable and precise early-stage disease diagnosis. By utilizing multiple modalities, it enables a comprehensive understanding of disease progression and infection within the human body. This approach enhances diagnostic accuracy and provides a more thorough analysis. Moreover, the utilization of multimodal data resolves diagnostic issues with greater precision and accuracy compared to singular-modality data [1]. Multimodal data is very important in treating critical diseases such as different types of cancer, brain tumor, tuberculosis, pneumonia, etc. Moreover, multimodality is also needed in complex clinical cases, where a peer consensus is needed for the diagnosis. Multimodal data fusion seeks to exploit the complementary, cooperative, and redundant features of different modalities to aid in the diagnostic process. The multimodality can be of both structured and unstructured types, with varying dimensions [2]. The use of machine learning (ML) models can assist in handling and revealing different patterns that would be concealed otherwise, for both structured and unstructured modalities [3].

Over the past 2 decades, machine learning models have demonstrated their potential in analyzing single-modality biomedical data. More recently, multimodal machine learning techniques have garnered significant interest in the field of biomedical data analysis. Deep learning, Convolutional Neural Networks (CNN), Long Short-Term Memory (LSTM), and Transfer Learning have extensively been employed to detect and predict diseases using single-modality data. With multimodal data being available, these methods extract features from various modalities, and further fusion techniques are used to create the most appropriate fused features for further analysis. However, several issues and challenges need to be considered before performing multimodal medical analysis. This review addresses the main challenges in multimodal medical analysis that are very important from the point of view of computational modelling. Since in biomedicine the accuracy and reliability of any AI-based model is very crucial, researchers should take these challenges as a prerequisite study. The review then also discusses various approaches proposed in literature to handle those challenges. Since in the field of biomedicine, the concept of AI-based multimodal diagnosis is new, the researchers have a lot of opportunities to provide targeted solutions for the biomedical data, which can be a new research direction.

The concept of multimodal diagnosis in medicine is very old, but AI-based multimodal diagnosis is appealing and attracting the involvement of computer scientists into medicine, thus making it as a multidisciplinary field. Additionally, AI-based multimodal diagnosis can somehow assist doctors and medical staff in the diagnostic process in rural or remote areas where there is a shortage of medical experts.

Our survey article is innovative as it provides a generalized version of all the major existing published surveys and research articles in the domain under study. We tried to cover all the aspects, which can be important for a researcher who would like to explore the research/knowledge in multimodal medical diagnosis. It covers multimodal diagnosis not only from a medical point of view but also from the computer science point of view, which allows readers to understand the concept and then apply it on real-time data.

### 1.1. Selection Criteria for the Articles Included in the Survey

**Figure 1:**
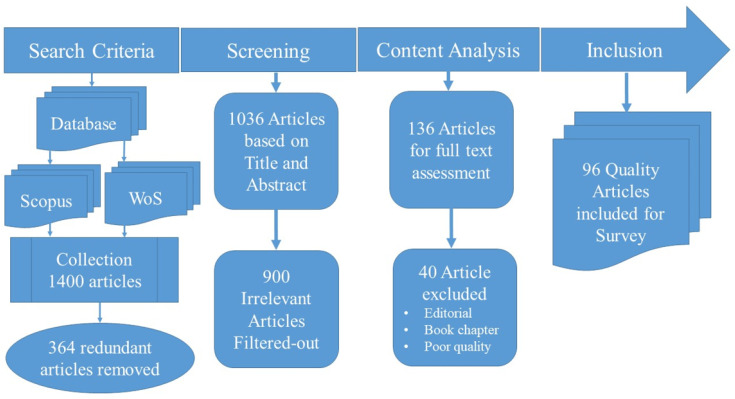
Selection criteria and process of article selection.

The process for searching, screening, and selecting the articles is shown in Figure 1. The reputed indexing databases such as Web of Science (SCI/SCIE/SSCI) and Scopus has been considered for the selection of the articles. Additionally, we limited our selection criteria to articles from prestigious publishers like Springer Nature, Nature, Elsevier, IEEE, and selected top-tier conferences such as NIPS (Conference on Neural Information Processing Systems) and ICML (*International Conference on Machine Learning*) to accumulate the literature. The list of publishers was determined by their reputation among the research community around the world after a long discussion. It was a qualitative approach rather than a quantitative one, and hence, there are no such measurable indicators available to report. The publishers that were selected are mentioned in the article, i.e., IEEE, Springer Nature, Elsevier, Nature publisher (including the *Nature* journal itself).

The search was based on keyword searches mentioning different combinations of the words “machine learning”, “deep learning”, “multimodal data”, “multimodal medical data”, and “multimodal medical diagnosis”. A total of 1400 articles published between 1990 and 2024 were collected as candidates, and 364 duplicates were removed. The remaining 1036 articles were thoroughly screened for title and abstract congruence with the aim of the present review study. The 900 articles that did not fall within the scope of the present study were excluded from further consideration. The 136 candidate articles were selected for detailed full-text evaluation and assessment, excluding 40 articles that were either editorials, book chapters, or of poor quality (unreliable methods and results). It is a known fact that the book chapters are the papers invited by the book editors and reviewed by them (Authors and editors know each other, so the review process is not fair). Since edited books are more focused on royalty earnings rather than quality publications, we decided not to include them. There is a big research community around the world who do not prefer to cite and refer articles that provide references to book chapters, so we decided to remove the book chapters.

### 1.2. Motivation and Contribution

In medical diagnosis, the physician relies on symptoms, medical history, medical imaging, various types of blood tests, histopathological tests, etc., to accurately diagnose the disease. The main reason is that some diseases are difficult to diagnose in the early stages, and therefore certain modalities may not show any signs of the diseases, but the combination of different modalities may show the presence of the disease. This can help the doctor to make a treatment plan to save the patient. The approach used by physicians motivates AI researchers to develop automated disease diagnosis models and methods using multimodal data to assist physicians. There are many existing publications on the use of AI-based automated diagnosis using single-modality data. However, very little work has been reported on multimodal data in the medical domain. The main problem is the unavailability of multimodal medical datasets for AI research. The data is available for sure, but the data is not organized and maintained. AI research is only possible if the data is organized, maintained, and prepared. Due to their heavy workload and high number of patients, doctors do not usually have time to collect data and organize it for research purposes, as their primary motive is to treat patients. Unfortunately, in hospitals, there are no dedicated department who maintain such data in digital form. There are research centers in some hospitals that collect information and store it in their database, but it is mostly imaging data; the clinical data is mostly hand-written and is available in paper form. And It is very difficult for it to be organized into one place, as previously, mostly imaging data was requested for AI-based research. Since multimodal data in medicine is a relatively new concept, research centers are trying to work on this.

In this review article, we address different issues related to the analysis of multimodal medical datasets such as different data sources that can contribute to the development of multimodal medical data, as well as different challenges in combining multimodal data and their representation, alignment, translation, co-learning, and fusion. Afterwards, different multimodal fusion approaches and related studies are discussed. The survey also provides a statistical overview on the number of papers published targeting multimodal medical analysis and the number of authors involved in the publication. A separate discussion is provided to highlight the existing review studies in the similar domain.

The rest of the article is organized as follows: Section 2 examines and analyzes important data sources in the medical field. Section 3 discusses challenges related to general multimodal data and proposes generic solutions to address them. Section 4 provides an overview of existing review studies and a statistical report on articles published on multimodal medical diagnosis. Section 5 discusses disease diagnosis and survival prediction with the use of multimodal data. It also lists available data sources that may prove useful. Section 6 details the possible ethical issues of using AI in multimodal medical diagnosis. Finally, Section 7 concludes by discussing limitations of the study.

## 2. Multimodal Medical Data Sources

**Figure 2:**
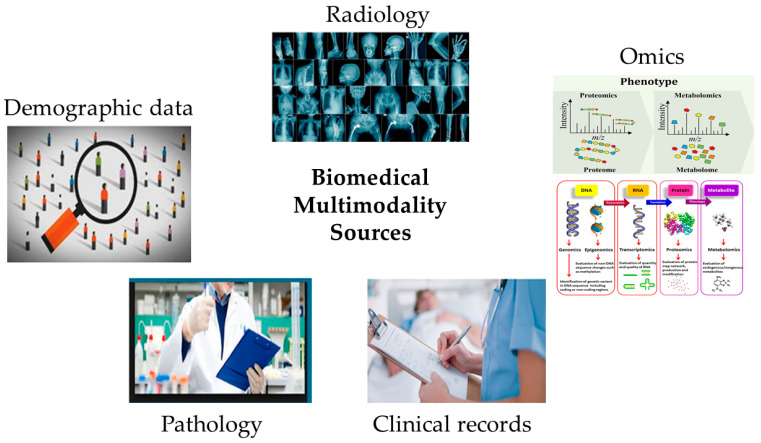
Leading multimodality sources in the biomedical domain.

Figure 2 depicts the main multimodal data sources in the biomedical field, including radiological, clinical, demographic, pathological, and omics data. The figure illustrates a few of the important different modalities available in the biomedical field. However, most of the time, the importance of any modality for diagnosis and treatment is purely subject to the doctor’s decision. Therefore, Figure 2 does not provide any temporal or other order for the availability and use of multimodalities, as their involvement in the diagnosis can be different for different types of diseases and entirely depends on the discretion of the physician. This section offers crucial details on these sources, while Table 1 showcases relevant studies that employed them for multimodal analysis in medical diagnosis.

### 2.1. Demographic Data

Demographic data is also very important from a medical point of view, as it provides important information that can help doctors in the diagnostic process. However, it must be utilized with other modalities to produce vital diagnostic results; otherwise, it becomes futile. Demographic data can provide valuable information regarding a patient’s ethnicity, age, origin, and medical history that can aid in answering a myriad of questions. Thus, it can be advantageous to incorporate demographic data in multimodal deep learning model training. Notably, scholars have combined demographic data with other modalities for multimodal fusion [4,5,6,7,8,9].

### 2.2. Clinical Records

Clinical records include patient information related to symptoms of the specific disease, past medical history, social and psychological factors, allergies, family history, and previous diagnoses [10]. Physicians record all the important factors relevant to the disease in the clinical record. Various clinical data is collected using wearable devices to measure heart rate, blood pressure, body temperature, and blood glucose levels [11]. Together, these parameters form the clinical record, which is important for diagnosis and prognosis of a disease. Clinical trials [12] provide another approach for gathering clinical records. However, conducting high-quality clinical trials is more time-consuming and expensive because it involves arranging and enrolling various participants to validate the efficacy of a therapy or medicine for a specific disease [4]. Xu et al. [5] utilized clinical records and cervigram images to develop a multimodal deep learning model for diagnosing cervical dysplasia.

### 2.3. Personalised ‘Omics’

Omics is a mechanism for quantifying and describing the biological molecules of human organisms. It includes terms like “genome”, “immunome”, “proteome”, “metabolome”, “epigenome”, and “microbiome” [13]. Omics plays a crucial role in the field of biomedicine by providing valuable information at the cell or tissue level for diagnosing different types of cancer at their initial stages. Each omic has distinct clinical and research significance in a specific disease. Furthermore, liquid biopsy samples obtained from blood and urine are extremely useful in the field of oncology [14]. Genetics and molecular markers are crucial in diagnosing cancer tumors [15,16]. Omics also help in comprehending polygenic risk scores [17] that provide details about the genetic and intricate traits of every human body. The integration of various omic types can enhance understanding of different effects, growth, and patterns of the disease in an individual. Yet, combining all types of omics poses a challenging task.

However, this integration is very important, because once done, omics data along with other modalities, such as electronic imaging and clinical data, will certainly increase the understanding of the growth and timely diagnosis of the disease, and further, to plan the post-diagnosis schedule and prevention strategies.

### 2.4. Pathological Records

Pathology records contain information regarding blood, urine, stool samples, and body tissues [6], each of which is crucial in determining the accurate diagnosis, stage, and progression of a disease. The records are categorized into four distinct groups, including anatomic, dermatopathology, forensic pathology, and laboratory medicine. Pathology records offer essential micro-level information about any disease. They are vital in medical diagnosis as they provide a precise understanding of the causal factors and disease outcomes. They are vital in medical diagnosis as they provide a precise understanding of the causal factors and disease outcomes. Due to these reasons, this modality is a leading component and should be included as part of a multimodal deep learning model. Using pathological images and electronic medical records (EMR), Yan et al. [7] developed a multimodal deep learning model for breast cancer detection. Similarly, Yao et al. [8] developed their own multimodal deep learning model by incorporating pathological images with cancer protein expression.

### 2.5. Radiology Data

Radiology involves diagnosing and treating medical injuries and diseases through medical imaging techniques like ultrasound, MRI, X-ray, CT scans, and more. These techniques are reliable sources for disease diagnosis and are one of the gold standards for automatic diagnosis. In recent decades, numerous research studies have created deep learning-based automated diagnosis methods that utilize medical imaging and radiology data [18]. Radiology imaging data is a favored modality for researchers seeking to fuse imaging data with other modalities. Thung et al. [9] and Li and Fan [19] utilized deep learning to carry out multimodal fusion with imaging data for Alzheimer’s disease diagnosis. Ou et al. [20] incorporated clinical images captured via smartphones with some metadata for multimodal fusion, although these images are not trustworthy for medical analysis and cannot be classified as radiology images. Additionally, the limited metadata offers no supporting information for the development of a multimodal fusion model.

**Table 1:** Different studies that used multimodality sources for medical diagnosis.

Reference Study	Modalities	Model	Dataset and Availability	Tasks
Xu et al. [5]	Cervigram, and clinical results of Human papillomavirus (HPV), Pap test	Convolutional Neural Network (CNN), Support Vector Machine (SVM)	Guanacaste project data of 10,000 women collected by National Cancer Institute (Public)	Cervical dysplasia diagnosis
Yan et al. [7]	Pathological images and Electronic medical records	CNN and Denoising Auto encoder	Patho EMR dataset (Protected)	Breast cancer classification
Yao et al. [8]	Pathological images and Molecular data	Deep correlation model	Public cancer survival dataset The Cancer Genome Atlas Program-Lung Squamous Cell Carcinoma (TCGA-LUSC) and TCGA-Glioblastoma Multiforme (TCGA-GBM) (Public)	Survival prediction on lung and brain tumor datasets
Thung et al. [9]	MRI images and demographic data	Multi-input-output neural network	Alzheimer’s Disease Neuroimaging Initiative (ADNI) Dataset (Public)	Alzheimer’s Disease diagnosis
Li & Fan [19]	MRI images and demographic data	Deep Recurrent Neural Networks	ADNI Dataset (Public)	Early diagnosis of Alzheimer’s disease
Ou et al. [20]	Photographic images and demographic and symptoms data	Encoder-based deep neural network	Programa de Assistȇncia Dermatolgica e Cirurgica (PAD-UFES-20) (public)	Skin lesion diagnosis
Xu et al. [21]	MRI images and clinical features	SVM, Logistic regression	Data of 101 HIV-infected patients (Protected)	HIV-associated neurocognitive impairment prediction
Zhao et al. [22]	ECG and facial video data	CNN	ULg Multimodality Drowsiness Database (DROZY) (Public) and Self-collected data (Protected)	Fatigue detection
Tiulpin et al. [23]	Knee X-ray images, Clinical data	CNN, Ensemble learning	Osteoarthritis Initiative (OAI) and Multicenter Osteoarthritis Study (MOST) (Public)	Knee osteoarthritis progression prediction
Li et al. [24]	Genomic data, pathological images	CNN	TCGA open source (Public)	Survival prediction in breast cancer patients
Xu et al. [25]	Different omics data	Deep Neural Network	Gene Network (Public)	Gene prediction
Fetit et al. [26]	Genomical, Retinal, and clinical data	Logistic regression (Lasso)	Genetics of Diabetes Audit and Research in Tayside Scotland (GoDARTS) (Public)	Diabetic cardiovascular disease prediction
Kayikci et al. [27]	Gene expression and clinical data	Attention-based CNN	Molecular Taxonomy of Breast Cancer International Consortium (METABRIC) (Public)	Breast cancer prediction
Steyaert et al. [28]	Histopathology images with gene expression	CNN	TCGA, Pediatric Brain Tumor Atlas (PBTA), Clinical Proteomic Tumor Analysis Consortium Glioblastoma Multiforme (CPTAC-GBM) (Public)	Brain tumor prognosis
Azher et al. [29]	Gene expression, histopathology, and DNA methylation	CNN	TCGA cancer data (Public)	Cancer prognosis

## 3. Need for Multimodality and Respective Challenges

**Figure 3:**
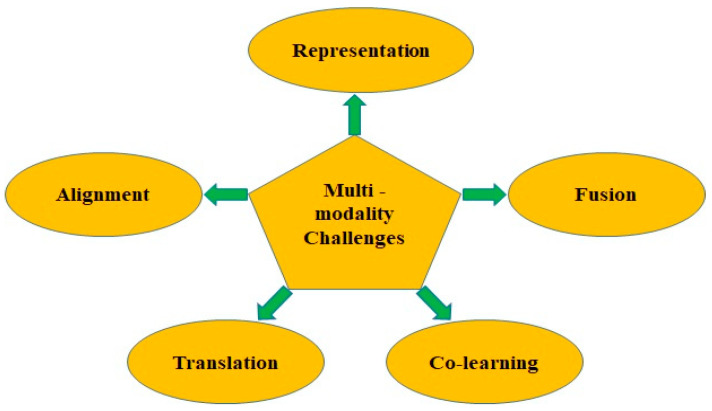
Five major multimodality challenges.

**Figure 4:**
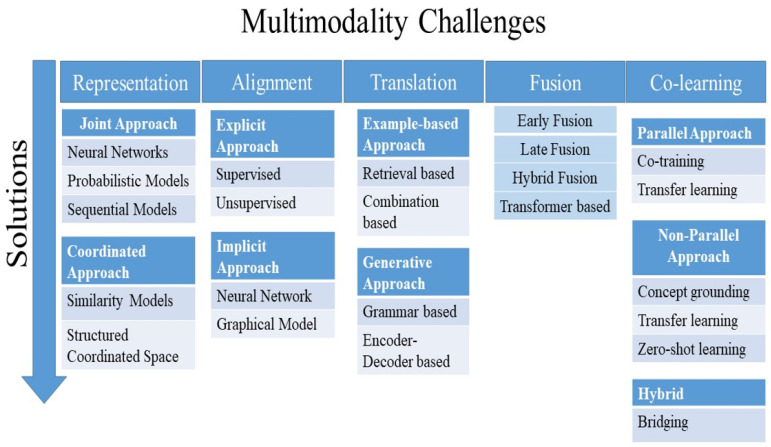
A brief illustration of multimodal challenges and solutions.

There are several challenges to address in multimodal machine learning. These challenges include multimodal representation, alignment, translation, fusion, and co-learning, as shown in Figure 3. Several solutions and approaches have been proposed by researchers to address and solve the challenges of multimodality, as shown in Figure 4 and discussed in the following subsections.

### 3.1. Multimodal Representation

Multimodal representation combines various modalities into a vector, tensor, or token format. The data can come from multiple sources and be homogeneous or heterogeneous, which affects the complexity of the coordinated representation. The data from various modalities may contain both redundant and complementary information, highlighting the need to represent this data in a clear and concise manner that can be easily processed. Challenges during multimodal representation include the presence of noise, missing data, and the heterogeneity of information. These challenges can be mitigated with joint and coordinated representation techniques. Table 2 presents significant studies that employed various techniques to project unimodal data into multimodal spaces, creating joint and coordinated representations.

**Table 2:** Important multimodal studies that proposed solutions to the multimodal representation problem.

Reference Study	Modalities				Joint Representation			Coordinated Representation	
	Text	Image	Audio	Video	Neural Networks	Graphical Models	RNN, LSTM	Similarity	Structured
Mroueh et al. [30]	-	X	X	-	X	-	-	-	-
Srivastava and Salakhutdinov et al. [31]	X	X	-	-	-	X	-	-	-
Frome et al. [32]	X	X	-	-	-	-	-	X	-
Kiros et al. [33]	X	X	-	-	-	-	-	X	-
Pan et al. [34]	X	-	-	X	-	-	-	X	-
Wu et al. [35]	-	X	X	-	X	-	-	-	-
Ngiam et al. [36]	-	X	X		X	-	-	X	-
Xu et al. [37]	X	-	-	X	-	-	-	-	-
Cao et al. [38]	X	X	-	-	-	-	-	-	X
Kim et al. [39]	-	X	X	-	-	X	-	-	-
Vendrov et al. [40]	X	X	-	-	-	-	-	-	X
Kahou et al. [41]	-	-	X	X	-	-	X	-	-
Zhang and Li [42]	X	X	-	-	-	-	-	-	X
Nicalou et al. [43]	-	-	X	X	-	-	X	-	-
Rajagopalan et al. [44]	X	X	-	-	-	-	X	-	-

#### 3.1.1. Joint Representation

In joint representation, multiple unimodal representations are mapped together in a multimodal space as Y_m_ = f(Y_1_, Y_2_,… Y_n_), where, Y_m_ is the multimodal representation computed using function f over a set of unimodal representations Y_1_,… Y_n_. The function mainly relies on the unimodal representations. Neural networks, graphical networks, and sequential networks are advanced methods for mapping several unimodal representations into multimodal ones. Creating a joint multimodal representation can be achieved using neural networks, graphical models, and recurrent neural networks. Advanced deep neural networks (DNN) and convolutional neural networks have been utilized in various unimodal representations for medical diagnoses. In recent years, researchers have utilized DNN and CNN to convert various unimodal medical datasets into multimodal representations [5,19,24,25,45].

***Neural networks*** are one of the important methods to represent unimodal data such as images, text, and electronic signals [46,47]. To map multiple unimodal data into a multimodal space, each unimodal data passes through several individual layers and then a hidden layer that projects each modality into a joint space [30,48]. Multimodal prediction can then be directly made using this multimodal joint representation. This process is known as multimodal representation learning or neural network-based multimodal fusion. To train neural networks effectively, advanced labeling of training data is required. Pre-training data using auto-encoder models, as described in [49], can be useful. This model can effectively train single modalities and can subsequently fuse multiple modalities together. If enough supervised data is not available, unsupervised auto-encoder models may also be used to pre-train neural networks effectively, which is particularly useful in multimodal representation. For medical diagnosis, some studies have used auto-encoder models to create multimodal representations [7,20].

***Probabilistic graphical models*** are another approach for joint representation. The design of graphical models employs latent random variables to create composite representations [46]. One such model is the Deep Boltzmann machine (DBM), which is utilized to model multimodal data. Each layer in the DBM adds increased complexity, leading to higher abstraction [50]. DBM specializes in the training of unlabeled data given its probabilistic representation [50]. DBMs have been applied to multimodal tasks by Srivastava and Salakhutdinov [31], where they coined the term “multimodal DBM”. DBMs have been utilized for audiovisual emotion recognition [39,51], audio- and skeleton-based gesture recognition [52], human pose estimation [47], and Alzheimer’s disease diagnosis [53].

***Sequential models*** are a slightly different approach for joint multimodal representation, which takes into consideration the present and previous state of the information during training. Recurrent neural networks (RNNs) and long short-term memory (LSTM) networks are excellent choices for sequence modeling tasks, according to [54]. Sequential models are broadly used in the natural language processing and computer vision fields for processing and analyzing sequences of words, images, or audio. RNNs and LSTM can effectively fuse multiple modalities, including audio, video, image, and text, to solve different tasks such as human behavior analysis [44], affect recognition [43,55,56], as well as anxiety and depression diagnosis [57].

#### 3.1.2. Coordinate Representation

Coordinate representation, rather than joint representation, learns individual representations for each sensory mode with a coordinated constraint, which is either a Euclidean distance or a partial order structured constraint. The coordinated representation of two sensory modes, m1 and m2, can be mathematically expressed as f(m1)~g(m2), where f and g are the projection functions of the sensory modes m1 and m2, respectively. Two methods for coordinating representations are similarity-coordinated and structure-coordinated models. It means that the two different modalities are projected into their own space but the coordination between them is provided by certain constraints. f(m1) is the projection of m1 modality into its space, and g(m2) is the projection of g2 modality in its space, and after projection the coordination is provided with certain constraints.

In ***similarity models***, the distance between different modalities is minimized in a coordinated space. For instance, in similarity models, the distance between the term “lung” and the corresponding image should be smaller than the distance between “lung” and an image of a “brain.” Such models enable linear mapping from textual to visual attributes, resulting in reduced spine distances between co-occurring annotations and images [58]. Some studies have developed similarity representation using neural networks [32], LSTM [33], and RNN [34,59]. In medical diagnosis, medical professionals can prepare medical images such as breast cancer mammography, chest X-rays, and CT scan images with proper annotations. The images can then be used to train similarity coordinated models for multimodal representation of medical modalities.

In ***Structured coordinated space***, additional structured constraint is enforced between modality representations. Canonical correlation analysis [60], kernel canonical correlation analysis [61], and deep canonical correlation analysis [62] are some examples of structured coordinate spaces for cross-modal retrieval. These unsupervised techniques are not parametric, but they may not be appropriate for handling large databases. Deep canonical approach is proposed to address scalability issues. These approaches capture the shared attributes of the modalities being represented. Therefore, they are beneficial when multiple modalities need to share information during multimodal representation.

### 3.2. Multimodal Alignment

Multimodality alignment can be defined as finding associations between small instances of different modalities. For example, given an X-ray image and a label such as the presence of a disease, the task is to find those areas in the image that indicate the presence of a disease. Multimodal alignment can be very useful for medical diagnosis if enough data can be prepared to train the model. In addition, multimodal alignment has a wide scope in multimedia retrieval. In general, it is possible to search for a movie by its title; but in multimedia retrieval, it is possible to search for a part or scene of a movie by text search. Multimodal alignment can be broadly classified into implicit and explicit. Table 3 shows some of the important studies on multimodal alignment.

**Table 3:** Important multimodal studies that proposed solutions to multimodal alignment problems.

Reference Study	Modalities				Implicit Alignment		Explicit Alignment	
	Text	Image	Audio	Video	Neural Networks	Graphical Models	Supervised	Unsupervised
Bojanowski et al. [63]	X	-	-	X	-	-	X	-
Kong et al. [64]	X	X	-	-	-	-	X	-
Zhu et al. [65]	X	-	-	X	-	-	X	-
Plummer et al. [66]	X	X	-	-	-	-	X	-
Mao et al. [67]	X	X	-	-	-	-	X	-
Malmaud et al. [68]	X	-	-	X	-	-	-	X
Tapaswi et al. [69]	X	-	-	X	-	-	-	X
Trigeorgis et al. [70]	-	-	X	X	-	-	-	X
Zhou and Torre [71]	-	-	X	X	-	-	-	X
Sjolander [72]	X	-	X	-	-	X	-	-
Vogel et al. [73]	X	-	X	-	-	X	-	-
Karpathy and Fei-Fei [74]	X	X	-	-	X	-	-	-
Xiong et al. [75]	X	X	-	-	X	-	-	-
Yu et al. [76]	X	-	-	X	X	-	-	-

#### 3.2.1. Explicit Alignment

Explicit alignment can be seen as the alignment between the instances of different modalities. The similarity metric is one of the important measures for explicit alignment, which can be learned or manually defined. Explicit alignment can be achieved by supervised and unsupervised methods, as explained below.

***Unsupervised Explicit Alignment*** does not require any labels to align the instances from different modalities [77,78]. In time series applications where multi-view time series are available, dynamic time warping (DTW) can be used as a similarity measure to align two different sequences [78,79]. An optimal match can be found using time steps and a similarity measure between two sequences [69]. Unsupervised alignment can also be achieved by using graphical models to align images with text in visual objects [80].

***Supervised Explicit Alignment*** is useful for labelled instances, as alignment is achieved by training the instances on similarity measure. Canonical time warping [63], canonical correlation analysis [66], and Gaussian mixture model [81] are some of the supervised techniques, which are inspired by unsupervised ones. Other popular approaches for supervised explicit alignment are based on deep learning models such as CNN and LSTM [66,67].

#### 3.2.2. Implicit Alignment

Implicit alignment is a latent supplementary task to support another task in the multimodal fusion process. Implicit alignment can be used as an intermediate step in machine translation and speech recognition. Implicit alignment is not involved in the direct alignment of two instances but provides latent support for data alignment during model training. Implicit alignment can be achieved by neural networks and graphical models.

***Neural network-based implicit alignment*** can be considered as an intermediate latent task that supports machine translation. To achieve this, Encoder-decoder or cross model can be substituted within a neural network to support the cause [74,82].

***Graphical models-based implicit alignment*** in general is not very effective for multimodal medical diagnosis if other than text modalities are included. Such models are commonly used for language translation or speech alignment with transcriptions [72,73]. In addition, it is also necessary to manually prepare the training data to map the features or instances between different modalities.

### 3.3. Multimodal Translation

Multimodal translation stands as one of the important and challenging tasks for multimodal fusion. Multimodal translation is about mapping the features from one modality to another to represent it in a more accurate way for further fusion. Cross-modal retrieval is one of the important tasks that can contribute a lot to multimodal medical data representation, such as translating image features into textual or numerical features or vice versa. It can be achieved by generative or example-based approaches. Table 4 shows some of the studies on multimodal translation.

**Table 4:** Important multimodal studies that proposed solutions to multimodal translation problems.

Reference Study	Multimodal Task			Generative Approach		Example-Based Approach	
	Text—Image	Image Captioning	Image Description	Retrieval Based	Combination Based	Grammar Based	Encoder-Decoder Based
Farhadi et al. [83]	-	X	-	X	-	-	-
Karpathy et al. [84]	-	X	-	X	-	-	-
Ordonez et al. [85]	-	X	-	X	-	-	-
Gupta et al. [86]	-	X	-	-	X	-	-
Kuznetsova et al. [87]	-	X	-	-	X	-	-
Lebret et al. [88]	-	X	-	-	X	-	-
Elliott and Keller [89]	-	-	X	-	-	X	-
Li et al. [90]	-	-	X	-	-	X	-
Mitchell et al. [91]	-	-	X	-	-	X	-
Kiros et al. [92]	-	X	-	-	-	-	X
Mao et al. [93]	-	X	-	-	-	-	X
Mansimov et al. [94]	X	-	-	-	-	-	X
Reed et al. [95]	X	-	-	-	-	-	X

#### 3.3.1. Example-Based Multimodal Translation

Example-based multimodal techniques depend on the training data, specifically the dictionary. The dictionary is a collection of different instances and their respective translations. Retrieval- and combination-based techniques are the categories of example-based multimodal translation.

***Retrieval-based translation*** tries to find the closest instance in the dictionary for the given sample and provides the translation as a result. Ordonez et al. [85] generated image captions using retrieval-based methods in a unimodal space. However, the main problem with unimodal space is that it requires a post-preprocessing step in which the re-ranking of the retrieved translations is done. This can sometimes lead to poor translations. As an alternative to the unimodal approach, the intermediate space can be used for similar comparisons [84]. Retrieval approaches in the intermediate semantic space perform better than the unimodal one.

***Combination-based translation*** can be seen as a step ahead of retrieval-based translation. The rules and conditions for these techniques are mostly heuristic or hand-crafted. These techniques combine the examples from the dictionary to provide them in a more meaningful way. Such an attempt was made by Gupta et al. [86] and Kuznetsova et al. [87]. They retrieved similar phrases from the dictionary that were relevant to the source input and then combined them to generate more useful translations. The combination-based translation can be very useful in preparing the medical dataset with medical images and their descriptions.

#### 3.3.2. Generative Approach for Multimodal Translation

In the generative approach, the idea is to construct a model to translate the unimodal source into the multimodal output. However, it requires an understanding of the source input modality along with the output sequence. The two generative approaches that could be useful in the multimodal medical domain are grammar-based and encoder-decoder-based.

***Grammar-based translation*** is a generative approach based on some predefined grammar concepts to generate a new modality. High-level features are first detected in the image or video modality, which are then combined based on a predefined grammar to generate the output modality. A similar approach was used in Li et al. [90] and Mitchell et al. [91], where the authors extracted the high-level features from the images, but different grammar rules were used to combine the features to generate the desired modality. However, the disadvantage of the grammar-based approach is that the generated modality is likely to be syntactically correct but may not be creative.

***Encoder-decoder-based translation*** is based on utilising the trained neural networks for multimodal translation. In this approach, the source modality is transformed into a feature vector by the encoder and then into the target modality by the decoder. A single pipeline is used to perform this task in an encoder-decoder fashion. The most popular ways to encode the source modality into vector features are convolutional neural networks [96], deep belief networks [97], and recurrent neural networks [98]. Furthermore, LSTM and RNN are the most commonly used approaches as a decoder [95].

### 3.4. Multimodal Co-Learning

The concept of co-learning is to support the features of the poor modality with the knowledge of the rich modality [99]. Co-learning is used during training of the model when one modality does not have enough resources to be learned by the model. Co-learning is not required for the testing phase of the model. Co-learning can be achieved in parallel, non-parallel, and hybrid modes. Parallel mode uses co-learning when there are not enough samples during training and generates more training samples [100].

The basic requirement for this technique is the availability of parallel data to support weak modalities. This could be very useful in medical multimodal data, which typically do not have sufficient samples in modalities such as clinical and demographic data. Other non-parallel co-learning methods can be used when modalities have nothing to share. Such methods are useful for object recognition, such as finding damaged or diseased nodules in lung images.

Transfer learning methods can be useful in both parallel and non-parallel data, as they are already trained on rich and large datasets. Both parallel and non-parallel approaches can be merged into a hybrid approach that uses a common modality to bridge the parallel and non-parallel modalities [101]. Co-learning is very important in several medical applications, such as the Internet of Medical Things (IoMT), where different modality sources are involved in data generation and analysis. Table 5 provide a summary on the co-learning issue.

**Table 5:** Important multimodal studies that proposed solutions to multimodal co-learning problems.

Reference Study	Co-Learning Approach					
	Parallel		Non-Parallel			Hybrid
	Co-Training	Transfer Learning	Concept Grounding	Transfer Learning	Zero Shot Learning	Bridging
Blum and Mitchell [100]	X	-	-	-	-	-
Krogel and Scheffer [102]	X	-	-	-	-	-
Ngiam et al. [36]	-	X	-	-	-	-
Shutova et al. [103]	-	-	X	-	-	-
Kiela and Clark [104]	-	-	X	-	-	-
Frome et al. [32]	-	-	-	X	X	-
Mahasseni and Todorovic [105]	-	-	-	X	-	-
Socher et al. [106]	-	-	-	-	X	-
Palatucci et al. [107]	-	-	-	-	X	-
Rajendran et al. [101]	-	-	-	-	-	X
Nakov and Ng [108]	-	-	-	-	-	X

### 3.5. Multimodal Fusion

Multimodal fusion is the important stage that greatly affects the outcome of the developed model. The choice of fusion techniques depends on the nature of the modalities considered. It is possible that some modalities are homogeneous in nature and have some instances in common. For example, X-ray and magnetic resonance techniques provide almost similar features, and in this case, an early fusion approach is applicable.

**Figure 5:**
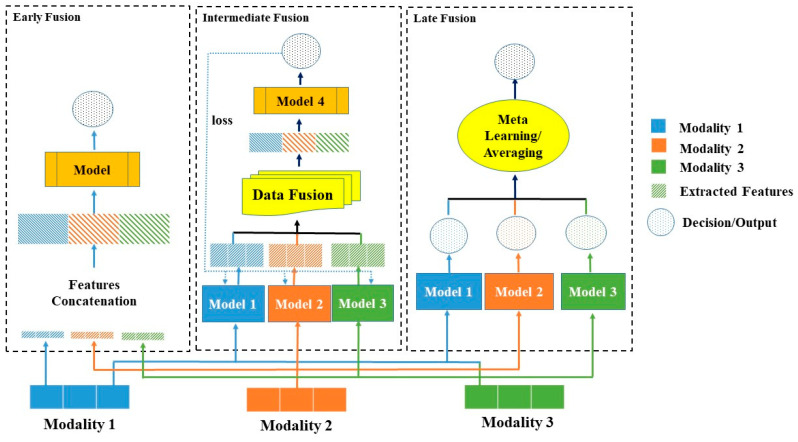
An illustration of early, late, and intermediate fusion.

In the case of heterogeneous modalities such as text and image, late or hybrid fusion is applicable. Figure 5 illustrates the different fusion strategies. Recently, transformer-based approaches were introduced in multimodal fusion, and they outperformed the traditional fusion approaches. Each late, intermediate, and transformer-based fusion will be discussed next. Table 6 shows various studies that used different fusion techniques in the medical field.

**Table 6:** Some important studies that used different fusion methods for the biomedical domain.

Reference Study	Fusion Strategy	No. of Modalities	Type of Modality	Technique Used	Targeted Disease	Conclusion
Xu et al. [5]	Early fusion	3	Image and non-image data	CNN, SVM	Cervical Dysplasia	Early fusion is usually not fit for heterogeneous modalities.
Abrol et al. [109]	Early fusion	2	sMRI and fMRI modalities (Scripts and Images)	SVM	Alzheimer’s Disease Progression	Decisions for fusion are made early on, restricting the ability to modify fusion techniques.
Boehm et al. [110]	Late fusion	3	Radiology and histopathological images and genomic data	CNN, LSTM	High-grade serous ovarian cancer	Late fusion results in a complex model to be compatible with knowledge obtained from various fusion techniques.
Hsu and Tseng [111]	Late fusion	2	Images and clinical data in vector form	HAC-LF based on CNN	Skin Cancer	It can be used only for hierarchical data.
Carrillo-Perez et al. [112]	Late fusion	5	RNA-Seq, miRNA-Seq, whole-slide imaging, copy number variation, and DNA methylation	SVM	Lung Cancer	System becomes very complex as the number of modalities increases.
Li et al. [113]	Early fusion	2	Images and clinical data	CNN	Brain Tumor	Inaccuracies in any data modality may affect the results badly.
Venugopalan et al. [114]	Early fusion	3	Images, genetic data, clinical data	Deep Models and Shallow models	Alzheimer’s Disease Stage	Feature extraction step is not performed end-to-end, which makes the model more complex.
Huang et al. [115]	Early, Hybrid, and Late fusion	2	Angiography images and EMR	Deep Neural Network	Pulmonary Embolism Detection	Late fusion outperformed the other two in this work.
Xu et al. [116]	Late fusion	3	Images, clinical data, lab tests data	KNN, Random Forest, and SVM	COVID-19 and Viral Infection	System becomes more complex to integrate high and low dimensional feature outcomes.
Xu et al. [117]	Late fusion	2	Images and clinical features	SVM	Neurocognitive impairment in HIV Patients	Output is affected by the quality of multimodality data.
Steyaert et al. [118]	Late fusion	4	Molecular data, histopathological data, clinical records	Deep Learning	Cancer	Data standardization and interpretability are the main challenges.
Sharma and Mandal [119]	Late fusion	5	MRI, fMRI, DTI, QSM, and MRS	SVM, ANN, DNN	Alzheimer’s Disease	Results are limited by the availability of appropriate data sources.
Jia and Lao [120]	Early fusion	2	fMRI and sMRI	SVM	Alzheimer’s Disease	End-to-end multimodal classification is not feasible.
Kumar et al. [121]	Late and Intermediate fusion	2	X-ray image and clinical data	CNN, LSTM	Lung Disease	Intermediate fusion performed better than Late fusion.
Zhou et al. [122]	Transformer based	2	Chest radiographs, laboratory test results	Encoder-decoder, MLP	Lung Disease	Unified representation outperformed the fusion approach.
Nguyen et al. [123]	Multi-agent transformer based	2	Radiographs, clinical data	Transformer	Alzheimer’s Disease	Transformer-based learning improves the performance.
Khadir et al. [124]	Transformer based	2	Clinical and radiology	Transformer encoder, MLP	Pulmonary Disease	Transformer-based learning improves the performance.

#### 3.5.1. Early Fusion

Early fusion is a technique that combines features from different modalities and fuses them instantly. Further, these fused features are fed into a model to perform prediction/classification. Early fusion does not pay more attention to feature learning because the feature concatenation is done at the initial level. Early fusion is useful when the different modalities under consideration contain homogeneous features; otherwise, the result may not be reliable. The advantage of this approach is that only one model needs to be trained, but as mentioned above, early fusion may not always provide a reliable outcome. Xu et al. [5] used early fusion to diagnose cervical dysplasia from image and non-image data using CNN and SVM. Abrol et al. [109] also used early fusion to predict the progression of Alzheimer’s disease using image and text data. Li et al. [113] used image and clinical data to classify brain tumor. The only problem with these studies is the heterogeneity of the modalities, which makes the result unreliable. Therefore, it is important to carefully investigate the modalities before using any fusion mechanism.

#### 3.5.2. Late Fusion

Late fusion is a decision-based fusion approach that makes the decision at the end. The separate models are trained for different modalities and then the output is fused using metaheuristic methods [121], averaging method [103], or voting approach [125]. Late fusion is quite useful when the modalities have nothing in common, as most suitable models can be trained separately for each modality. Then, any approach is used to fuse the outputs and obtain the results. The main drawback of late fusion is that low-level interactions between modalities are ignored, which may make the model unreliable, at least for medical diagnosis.

Boehm et al. [110] developed a late fusion model using clinical images and genomic data. Genomic data is complex in nature because it has its own different types. Therefore, late fusion is a good choice, but the correlation between imaging and genomic data is ignored. Hus and Tseng [111] predicted skin cancer with a late fusion approach using clinical and imaging modalities. Carrillo-Perez et al. [112] used late fusion with five different modalities, which made the model too complex. It is desirable that low-level feature learning must be done when multiple modalities are involved in multimodal fusion, which is a drawback of late fusion.

#### 3.5.3. Hybrid/Intermediate Fusion

The hybrid fusion approach considers the benefits of early and late fusion. Hybrid fusion allows one to learn the low-level dependencies between the features of different modalities. One approach is to train separate models on different modalities and then use the obtained features as input to the fully connected neural network model. The neural network then trains itself again on new combined features to perform prediction/classification. In addition, back-propagation can be used to update the weights of the network to improve the accuracy/loss ratio.

Kumar et al. [121] compared the late and intermediate fusion models on lung disease data with clinical and imaging modalities. They found that hybrid fusion outperformed the late fusion approach. Huang et al. [115] developed and compared all early, late, and intermediate fusion for pulmonary embolism detection. They found that the late fusion model outperformed the other two. Therefore, it is unclear which fusion technique is best. However, it is recommended to develop all fusion before making a conclusion on a specific dataset.

#### 3.5.4. Transformer-Based Fusion

As discussed in previous sections, low-level feature learning is very important to develop a successful multimodal fusion model. Vashwani et al. [126] proposed a transformer model based on the attention mechanism for machine translation. Furthermore, the model was used in natural language processing, computer vision, and multimodal fusion. Transformer architecture uses an encoder-decoder mechanism to process the input features and generate the new features that have learned the low-level interactions between modalities [126].

Zhou et al. [122] developed a transformer-based learning model that represents the features of image and clinical modalities in a unified form. They suggested that a token-based approach is more suitable for text and image modalities for unified feature fusion. Nguyen et al. [123] used two separate transformer attention models for Alzheimer’s disease prediction, one for image modality and the other for fusing clinical features with image features. Their model outperformed the baseline models. Khadir et al. [124] developed large-scale transformers with the goal of providing scalability and computational efficiency to handle large datasets. They used one transformer encoder to handle clinical and imaging data and then another transformer to fuse the two features. Khadir et al. [124] claimed that the model is scalable and computationally efficient for large multimodal datasets.

## 4. Overview of Existing Studies and Statistics

### 4.1. Existing Review Studies Published on Multimodal Medical Diagnosis

Recently, Shaik et al. [127] published a survey paper addressing multimodal fusion for smart healthcare. They focused on algorithmic approaches for handling multimodal data, such as feature selection, natural language processing, fusion of data using machine learning and deep learning methods, and rule-based systems. Several challenges towards smart healthcare were also addressed. Mohammad et al. [128] also discussed several issues on multimodal signal fusion for smart healthcare systems. The survey paper was more IoMT oriented and addressed several key issues related to IoMT applications and challenges for smart healthcare.

Pei et al. [129] addressed several medical applications of multimodal deep learning in their review paper. The key finding of the review paper was the detailed discussion and thorough comparison of existing general pertained multimodal fusion algorithms and medical multimodal fusion algorithms. Lawonn et al. [130] addressed and discussed various key challenges in multimodal data visualization. The authors discussed several unresolved issues of multimodal data visualization to provide researchers with a new direction to explore. However, their discussion was medicine oriented, and they did not discuss AI-enabled multimodal visualization. Acosta et al. [18] presented analytical and technical challenges of multimodal biomedical AI. The authors discussed several applications and opportunities for multimodal analysis in clinical trials, personalized medicine, pandemic surveillance, and smart healthcare. Amal et al. [131] discussed the application and scope of multimodal data and machine learning with respect to cardiovascular healthcare. The authors also briefly discussed the challenges of multimodal data fusion.

Gao et al. [132] discussed various deep learning aspects for multimodal big data. The review study was not directly concerned with medicine, but the techniques discussed were potential candidates for multimodal medical diagnosis. Biessmann et al. [133] discussed in detail the neuroimaging aspect of human brain activity. The authors stated that combining neuroimaging modalities could reveal some hidden technical and physiological facts. These facts can further be useful in developing AI-based multimodal solutions to address and understand the low-level peculiarities to improve brain health.

Cai et al. [134] presented a smart healthcare systems-oriented review paper in which they covered various approaches of multimodal data fusion for smart healthcare. The review paper also covered several other aspects of smart healthcare systems such as the applicability of cross border association, healthcare-oriented semantic perception, alignment, etc. Huang et al. [135] presented a review on deep learning-enabled multimodal fusion of electronic records and medical imaging. The authors stated that early, joint, and late fusion approaches are deployable for multimodal fusion of imaging and clinical data. They also described some guidelines to consider before deploying any fusion approach for medical multimodal data.

Stahlschmidt et al. [136] mentioned that biomedical data is increasingly becoming multimodal, providing a valuable source of hidden information that is not accessible through single-modality approaches. Deep learning techniques are able to fuse the multimodal data and capture the complex relationship between the modalities. The authors also mentioned that transfer learning is a good solution for multimodal medical big data. Kline et al. [137] also discussed various machine-learning techniques for multimodal data. They discussed different types of medical data, fusion techniques, along with the limitations of multimodal fusion approach.

### 4.2. Statistics of Studies Published on Multimodal Medical Diagnosis

**Figure 6:**
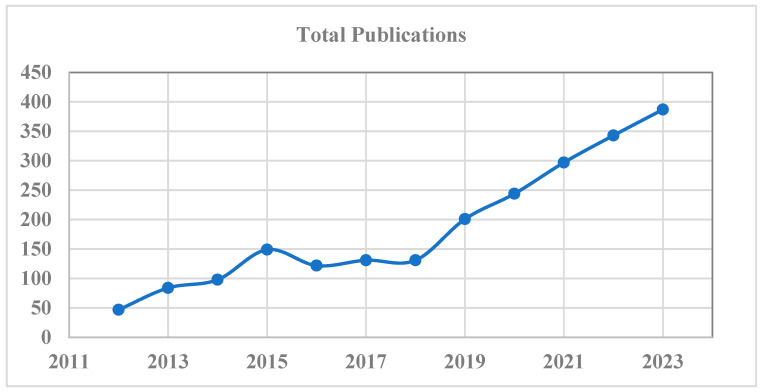
Number of articles published on multimodal medical diagnosis (2012–2023).

**Figure 7:**
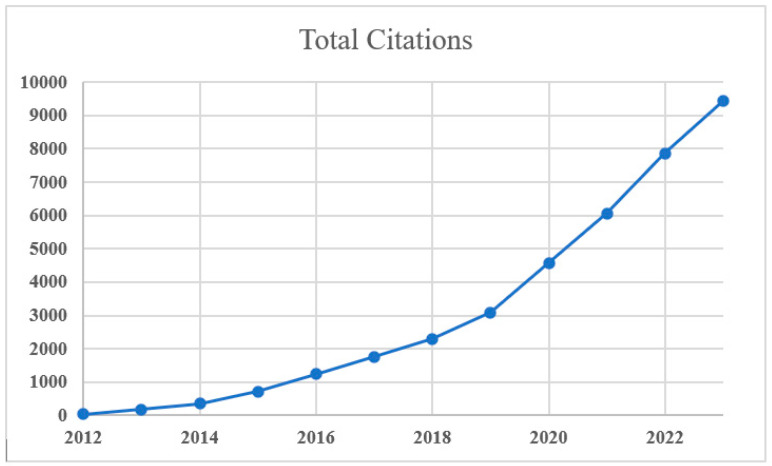
Number of citations on articles published on multimodal medical diagnosis (2012–2023).

We conducted a statistical analysis to examine the prevalence of published studies on multimodal medical diagnosis. Our analysis considered articles on “multimodal medical diagnosis” published between 1 January 2012 and 25 November 2023. The term “multimodal medical diagnosis” considers both disease prediction and classification studies. The title and abstract were selected as search criteria on the online dimension web application [138] to identify relevant publications. Figure 6 illustrates the annual publication count for multimodal medical diagnosis research. There has been a noticeable rise in the quantity of publications pertaining to multimodal medical diagnosis over the past 5 years, indicating that this topic has garnered substantial interest within the research community. Figure 7 provides an illustration of the number of citations attributed to published articles on multimodal medical diagnosis, highlighting an increasing trend since 2012. This correlation between the number of citations and publications implies that a surge in published articles has resulted in a higher number of citations.

**Figure 8:**
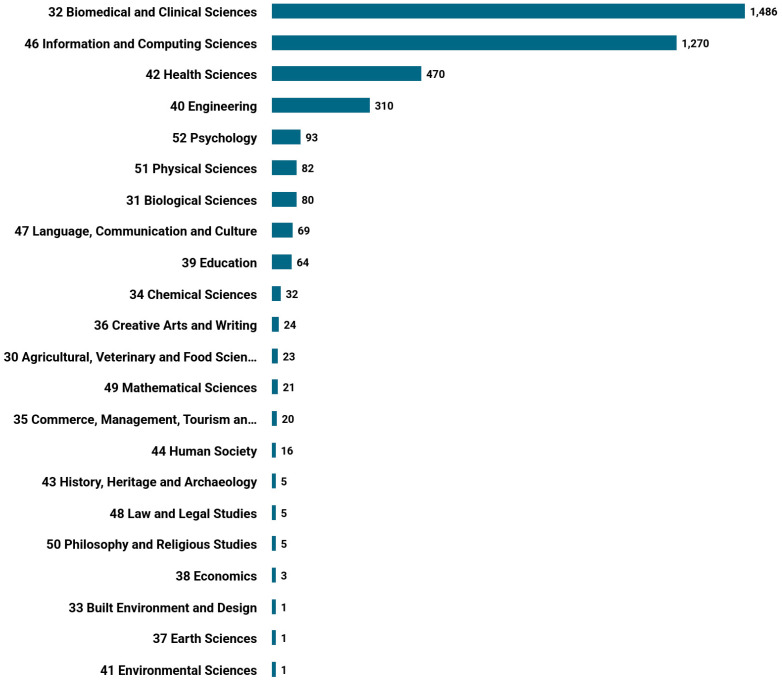
Research category-wise distribution of articles in multimodal medical diagnosis.

We also analyzed the contribution of authors from diverse research fields in multimodal medical diagnosis. Our study revealed that roughly 1420 publications belong to the biomedical and clinical science field, followed by 836 publications in information and computing sciences. However, the involvement of other categories is minimal. See Figure 8 for a categorical breakdown of multimodal medical diagnosis articles.

**Figure 9:**
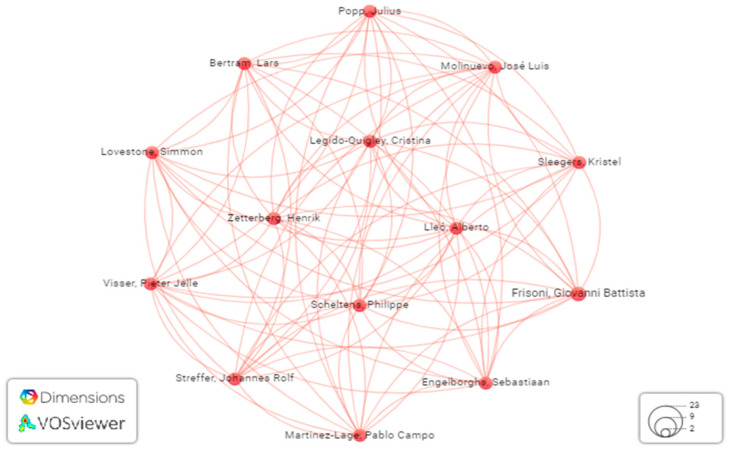
Relativeness of researchers based on co-authored publications.

**Figure 10:**
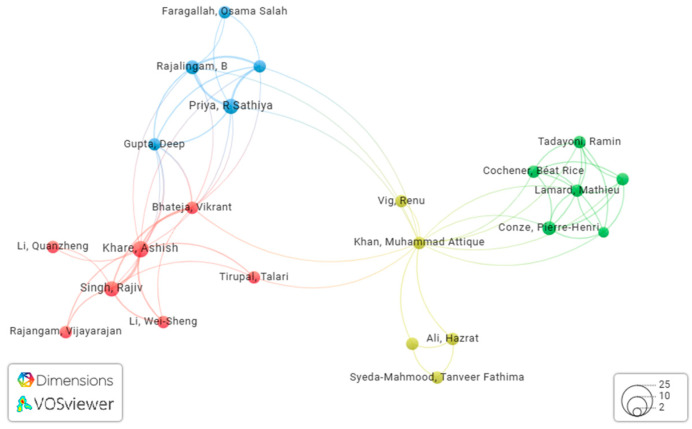
Relativeness of researchers based on the citations.

Multimodal medical diagnosis is a multidisciplinary research endeavor. As such, we examined the correlation of co-authorship among publications on multimodal medical diagnosis. Figure 9 displays the interdependence among authors who shared co-authorships in multiple research publications on multimodal medical diagnosis. Highly cited studies were examined to determine the correlation. Figure 10 shows the relationships among authors based on citations. This information is beneficial for readers as it allows them to quickly access the author’s scientific profile and related articles in the field. Since this field is new, researchers might be interested in following certain active researchers in multimodal medical data analysis using AI. Hence, Figure 10 can be useful in identifying certain active researchers in the field in order to follow their work and approach instead of searching for the articles on Google Scholar.

It can be concluded that multimodal medical diagnosis is an essential and expanding area of research. Due to its multidisciplinary nature, researchers from the fields of medicine, artificial intelligence, electronics, and computer science can collaborate to develop an efficient solution and enhance the quality of smart healthcare.

## 5. Multimodal Medical Diagnosis: Disease Diagnosis, Survival Prediction, Dataset Availability, and Future Directions

Disease classification and survival prediction are two different but important concepts in medicine [8]. The first one confirms the presence of the disease, and the second one deals with deciding the correct treatment plan and ensuring the progress of the treatment [24,139]. In the following two subsections, we will outline some important studies that have used multimodal deep learning for disease diagnosis and survival prediction.

### 5.1. Multimodal Disease Classification/Prediction

Early diagnosis of diseases is very important in the medical field because several diseases have high mortality rates and low survival rates. Initially, medical imaging techniques were widely used for early diagnosis of several diseases such as pneumonia, skin cancer, brain tumor, and breast cancer [140,141,142,143]. However, the confirmed diagnosis depends on other factors such as laboratory analysis and pathological examination [121]. Mishra et al. [144] developed a deep learning-based model to classify breast cancer using ultrasound and B-mode images. The authors used the clinical breast US dataset for the study. Since both modalities are image-based, the early fusion approach was used for classification.

Yan et al. [7] develop a hybrid fusion network on clinical and histopathologic modalities that extracts features from both modalities. In addition, the clinical features were enhanced using an autoencoder to match the imaging features extracted using convolutional networks. A fully connected neural network was then used to train on the concatenated features to perform the classification. The authors claimed that their model achieved high performance on multimodal data than on single-modality data.

In [114], Venugopalan et al. attempted to develop a multimodal framework for early detection of Alzheimer’s disease. The authors used MRI images, clinical, and genetic data to develop the model and classify patients into three categories. They found that deep networks outperformed shallow machine learning models on multimodal data. Dwivedi et al. [144] proposed a multimodal diagnosis method for Alzheimer’s disease using MRI and positron emission tomography (PET) images. The authors used a Harnessing Demon algorithm with DWT to optimally fuse the images.

Furthermore, the ResNet 50 network was used to extract the features, and classification was done using SVM. The authors claimed that their model achieved 97% accuracy. Zhang and Shi [145] used multimodal feature fusion for Alzheimer’s disease diagnosis by exploiting both early and late fusion on MRI and PET images in a hierarchical manner. They used attention mechanisms for feature extraction. Furthermore, the model was tested on the ADNI dataset, and it outperformed the state-of-the-art methods. Moreover, all these attempts were aimed at developing a multimodal fusion model for disease diagnosis and prediction. It is recommended to involve medical practitioners, especially cancer experts, to understand the smallest peculiarities of serious diseases, which can play a big role in developing AI-based fusion models for medical diagnosis.

### 5.2. Multimodal Survival Prediction

Another important aspect of medical diagnosis is predicting a patient’s survival. Effective diagnosis and timely treatment are key to a patient’s successful recovery. Therefore, besides early diagnosis of the disease, survival prediction is also the point of interest for AI and medical researchers. Survival analysis can be seen as a technique to evaluate the progress of an event over time, e.g., cancer survival prediction and disease recurrence probability. The existing methods for survival analysis fall into parametric, non-parametric, and semi-parametric. Kaplan-Meier [146] and Nelson-Aalen [147] are non-parametric methods; Cox regression [148] is a semi-parametric method; and Deep Hit [149], Cox-time [150], and CoxCC [139] are parametric methods for survival analysis. In multimodal scenarios, these statistical models can be used in the final step for decision-making. Wu et al. [151] developed a multimodal fusion-based approach for survival prediction in lung cancer patients. They used non-small-cell lung cancer data with two different modalities, i.e., clinical records and 3D CT images. 3D CT image features were extracted using ResNet, while clinical records were processed using One-Hot Encoder. Furthermore, clinical and image features were embedded and a fully connected neural network with one hidden layer was used for survival analysis. The authors claimed that their results outperformed the other baseline models.

However, we believe that other fusion strategies may improve the results. Vale-silva and Rohr [152] also attempted to predict long-term cancer survival and developed MultiSurv. Clinical, omics, and imaging modalities were used to predict survival. They applied the model to 33 cancer types and claimed that the model outperformed several baseline models on various time-dependent metrics. Hao et al. [153] used several types of genetic data to address the problem of cancer heterogeneity. They extracted features from multiple genetic data and concatenated them using a deep feature learning network for survival prediction. They confirmed that the model achieved better performance in long-term cancer survival prediction than the related works. Fan et al. [154] used clinical and multi-omics data for pan-cancer survival prediction. The authors used attention-based multimodal fusion to fuse clinical and omics data. They also used a fully connected neural network for survival prediction. Their model achieved higher performance than existing ones on the majority of cancer data.

Fu et al. [155] developed a graph-based network to fuse imaging mass cytometry (IMC) images with patient variable data (gene indicators and clinical parameters). They tested their model on two different breast cancer datasets and found that the model outperformed the existing models in predicting the survival of breast cancer patients. The attempt was indeed good, but the survival prediction of breast cancer on medical grounds is around 89.6% [151]. Therefore, we recommend that the authors must also test the model on lung cancer survival prediction, which has a very low survival rate than other cancers [151]. It is obvious that survival prediction is very important for cancer diseases, as it plays a vital role in planning the accurate treatment and monitoring the progression of the disease.

### 5.3. Available Datasets for Multimodal Medical Diagnosis

The availability of datasets is the basic requirement for any multimodal medical diagnosis using deep learning [156]. The researchers and medical practitioners are making constant efforts to prepare multimodal medical datasets. Several studies have used multimodal data for medical diagnosis and survival prediction. Some of the datasets are publicly available, some are proprietary but available on special request, and some are private and not available for use. Table 7 provides information about some of the important and reliable datasets, their availability and source, possible tasks within the data, and diseases and modalities in the dataset. Interested readers can explore these datasets for further detailed understanding and knowledge.

**Table 7:** Some important multimodal medical datasets.

Dataset	Availability	Data Source	Possible Task	Type of Disease and Modalities
HAIM multimodal dataset	Protected (Available upon Request)	https://physionet.org/content/haim-multimodal/1.0.0/ accessed on 16 September 2024	Disease diagnosis and survival analysis	Many pulmonary diseases, eye diseases; X-ray imaging, clinical and laboratory tests
The Cancer Genome Atlas (TCGA)	Public and partially Protected	https://www.cancer.gov/ccg/research/genome-sequencing/tcga accessed on 16 September 2024	Disease diagnosis and survival analysis	33 cancer types; Imaging, clinical, and molecular data
Multi-Omics Cancer TCGA	Public	http://acgt.cs.tau.ac.il/multi_omic_benchmark/download.html accessed on 16 September 2024	Disease diagnosis, survival analysis, Omics clustering	Cancer; multi-omics
Breast Cancer Multimodal Data (AI research centre in SMC)	Private (May be available on request)	Source of the article: https://www.nature.com/articles/s41598-021-98408-8 accessed on 16 September 2024	Pathology response prediction	Breast Cancer; Clinical data and imaging
NSCLC Cancer Patient Data (TCIA)	Public	https://jksronline.org/ViewImage.php?Type=TH&aid=660600&id=T6&afn=2016_JKSR_80_2_176&fn=jksr-80-176-i006_2016JKSR accessed on 16 September 2024	Survival prediction	Non-small-cell lung Cancer; 3D X-ray imaging, clinical data
METABRIC	Public	https://idr.openmicroscopy.org/ accessed on 16 September 2024	Disease diagnosis, Survival prediction	Breast Cancer; Imaging and patient data
BASEL	Public	https://zenodo.org/records/3518284 accessed on 16 September 2024	Disease diagnosis, Survival prediction	Breast Cancer; Imaging and patient data
PathoEMR	Private (Public link is not found)	https://bmcmedinformdecismak.biomedcentral.com/articles/10.1186/s12911-020-01340-6 accessed on 16 September 2024	Disease diagnosis	Breast Cancer; Imaging and clinical data
ADNI Dataset	Public	https://adni.loni.usc.edu/ accessed on 16 September 2024	Disease diagnosis, Disease progression prediction	Alzheimer’s Disease, Imaging and clinical
PAD-UFES-20	Public	https://data.mendeley.com/datasets/zr7vgbcyr2/1 accessed on 16 September 2024	Disease diagnosis	Skin disease

## 6. Ethical Issues of Using AI in Multimodal Diagnosis

**Figure 11:**
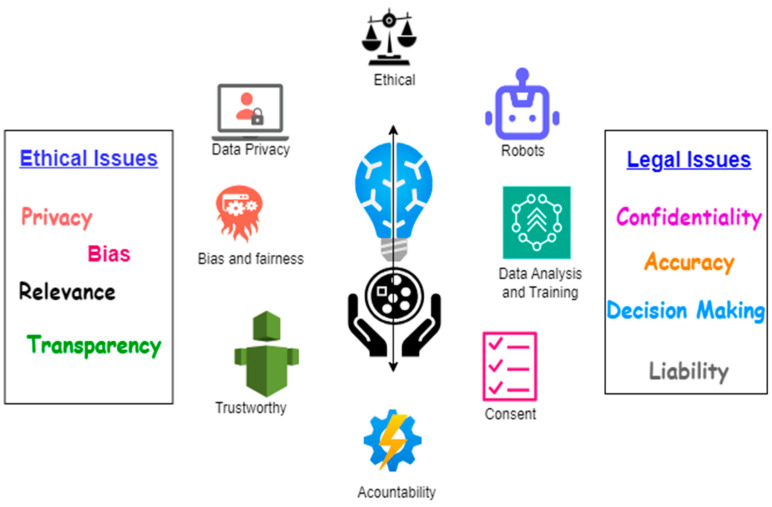
AI in Multimodal Medical Diagnosis System: Ethical and Legal Issues.

AI applications in multimodal medical environments have brought about significant changes in the medical field. Multimodal diagnosis requires data medical imaging, laboratory diagnosis, treatment methodology, provision of preventive and precision medicine, extensive analysis of data, etc. [157,158]. However, incorporating AI in the multimodal medical domain encounters a multitude of ethical and legal hurdles. Despite notable advancements in AI within communities and its contribution to enhancing treatment protocols, its reach remains limited across societies. It is important to acknowledge the array of challenges including ethical issues, privacy concerns, sensitive data protection, informed patient consent, etc., that arise while utilizing AI in a medical environment. Therefore, it is important to consider all four pillars of medical ethics—autonomy, beneficence, non-maleficence, and justice—before integrating AI and the healthcare system. A brief view of these ethical and legal issues is presented in Figure 11. We explain each of these ethical issues in concern to medical pillars one by one in detail as follows:

### 6.1. Ethical Issues When AI Meets Data Handling

In a multimodal environment for disease diagnosis, data is collected from heterogeneous sources like images, clinical, laboratories, etc. Some forms of data are more sensitive and confidential to be shared. The incorporation of AI in the healthcare sector sparks concerns regarding potential inaccuracies and risks of data breaches. Artificially intelligent systems like robots come up with the risk of being hacked and appropriate sharing of sensitive information, leading to unauthorized access and misuse [158]. Additionally, within the realm of AI, social media plays a crucial role in disseminating health news and medical advice, especially in pandemics. These aspects of AI seem beneficial. However, ensuring the privacy and security of patient data remains a point of concern, particularly when AI algorithms are used for mining these data and publishing some sensitive patterns [157,158]. Robots collect sensitive data and are susceptible to hacking [157]. This raises the question not only on the consent policy but also on eroding trust, leading to legal ramifications.

### 6.2. Ethical Issues with AI in Terms of Bias and Fairness

It has been observed in many real-time cases that AI models can embed and deploy human and social biases. AI models are trained using data that contains human decisions or may reflect the second-order effects. The method used for data collection and its use can contribute to bias in AI models. The more we rely on decisions made by AI-based systems in healthcare, it is quite important to ensure that they are made ethical and free from unjust biases [159]. The AI models that have been trained in one region of the world get biased according to environmental, population, and medical expert data associated with the region and may not work well when used in different regions. Therefore, they may not be able to treat patients efficiently and suffer from sample biasing. Further, the AI model may lose accuracy due to annotation biasing and cause discriminatory decisions. Additionally, AI models are like old doctors, i.e., more practice helps them to gain more knowledge and experience, which in turn helps them to make good decisions. The training time of AI models is crucial, especially in the healthcare sector. Two models may differ in their expertise if they have different training times. This may lead to temporal biasing [160] and become unfair in making decisions.

### 6.3. AI and Transparency Ethics

AI-based data analysis and decision-making act as a black box to the customers. The information about decisions and analyses made by AI algorithms is not clear to the clients. Ethical issues in terms of transparency arise in terms of recommendations or medical assistance [161]. For example, IBM Watson for Oncology [162] uses AI algorithms to access information from patients’ medical records and assist doctors in exploring cancer-treatment options for their patients. However, after a year it was reported unsafe and was criticized [163] because IBM Watson was using synthetic data instead of real patient data. Therefore, it is quite important what data these AI systems are trained with. Transparency must be provided to the end users so that trust over recommendations can be built. However, disclosure of data again causes data privacy threats. The recommendations generated from block box AI systems raise concerns, and it is challenging to understand the way to provide transparency in this context [164].

### 6.4. AI and Absence of Responsibility

Nowadays, in a medical environment, most of the critical decisions are taken by AI-driven intelligent systems. These decisions should complement human decisions. Due to complex data analysis and interactions, these decisions must be supervised very carefully by humans. AI-driven decision-making raises the question of responsibility. Unlike doctors, AI systems are not obligated by the law of accountability for any action or decision [165]. No clinician can appropriately justify the actions taken by AI systems. This absence of accountability gives rise to apprehensions regarding the potential safety risks associated with employing unverified or invalidated AI systems in a clinical environment [166]. According to the Association for the Advancement of Artificial Intelligence (AAAI), it is imperative to access and validate AI systems thoroughly before their implementation. It is critically important to test, measure, and assess the dependability, performance, and safety of such robotic systems. If AI-driven systems remain immune to accountability measures, their involvement in roles requiring human care may be compromised. Managers overseeing AI systems usage must ensure that physicians cannot evade responsibility by shifting blame onto the AI systems.

### 6.5. AI, Healthcare, and Legal Issues

AI systems in the healthcare industry are playing a critical role in medicine, clinical, as well as drug research. It is quite important to have legal rules and regulations for the usage of intelligence systems in the industry. Unlike medical professionals, there are still no clear rules and legal actions associated with any analysis, outcome, or decision-making done by these intelligent robots or systems [167]. Therefore, developing robust regulation for AI and robotics is of prime importance. There is no accountability or liability framework defined for any robotic error or decision-making. Clear responsibility must be allocated among manufacturers, healthcare institutions, and professionals [168]. Defining ownership and rights in AI-generated medical data is complex. There is no existing ethical framework that prioritizes data sharing and preserves data privacy while using intelligent systems. This again goes against the data security law of patients and is a critical legal issue that arises while using intelligent systems in medicine.

## 7. Conclusions and Limitations

Multimodal medical diagnosis combines multiple sources such as medical imaging, laboratory and pathological tests, and clinical records to confirm the presence of the disease. Due to the unavailability of experts in various rural and suburban areas, the requirement of multimodal automatic diagnosis is very important. Deep learning approaches are very effective in exploiting single-modality data. However, to deal with multimodality data, several issues and challenges need to be considered. This review paper provides all the relevant important information for the multimodal medical community, which will benefit them in furthering multimodality medical research. We conclude that automated multimodal medical diagnosis can be a life-saving effort in medicine. Readers, students, and researchers who are interested in exploring multimodal data analysis in the field of biomedicine can benefit from this review, as it provides a comprehensive detailed analysis of the various challenges, available datasets, and relevant research articles in summarized form. Readers may use this knowledge and guidance available in this review to brainstorm new ideas to improve the present methods and provide a more accurate multimodal medical analysis in the future.

This review does not cover all aspects of multimodal data beyond the medical domain. We have included and addressed only those concepts that are important from a medical diagnostic point of view. We believe that this article will surely benefit academicians and researchers who want to explore their interest in multimodal medical diagnosis.
